# Explainable Ensemble Machine Learning for Predicting Deposition Characteristics in Advanced Additive Manufacturing

**DOI:** 10.3390/mi17060663

**Published:** 2026-05-27

**Authors:** Sandeep Jain, Pradyumn Kumar Arya

**Affiliations:** 1 School of Materials Science and Engineering, Yeungnam University, Gyeongsan 38541, Republic of Korea; 2 Department of Mechanical Engineering, Indian Institute of Technology Delhi, Hauz Khas, New Delhi 110016, India; pradyumn.mec@gmail.com

**Keywords:** machine learning in manufacturing, advanced additive manufacturing, deposition characteristics, SHAP analysis, ensemble machine learning

## Abstract

In advanced manufacturing processes, precise deposition behavior prediction is crucial for process parameter optimization. In order to forecast significant deposition responses such as bead width (w), bead height (h), energy input (EI), and volumetric input (VI) based on process parameters like laser power (P), travel speed (v), and wire feed rate (fw), seven different machine learning (ML) models were developed in this study, including Random Forest (RF), Gradient Boosting (GB), Extreme Gradient Boosting (XGB), LightGBM (LGBM), Extra Trees (ET), Support Vector Regression (SVR), and Elastic Net (EN). The predictive power of all models was assessed using the mean absolute error (MAE), root mean square error (RMSE), and coefficient of determination (R^2^). The discoveries showed that ensemble models performed better than traditional ML techniques. The GB model performed the best overall, followed by the XGB model, which showed strong generalization and high prediction accuracy across training, validation, and testing datasets. Additionally, computational efficiency research discovered that the GB model holds a moderate model size and practically quick training time, making it suitable for real-world application. The robustness of the chosen model was supported by a paired *t*-test, which verified that the performance differences between the GB model and other models are statistically significant (*p* < 0.05). Furthermore, the impact of input parameters on the anticipated responses was interpreted using SHAP (SHapley Additive exPlanations) analysis. The interpretation results exhibited that while wire feed rate mostly affects volumetric deposition behavior, laser power and travel speed are the key parameters monitoring energy input and bead geometry. The GB and XGB models were used to forecast deposition reactions using specific process parameters, and the predictions were compared with experimental findings in order to further confirm the predictive power of the developed models with better performance. Overall, the results display that this study, in combination with ensemble boosting models, offers a reliable framework for understanding complicated relationships between deposition features and processing parameters, providing insightful information for process optimization in advanced manufacturing applications.

## 1. Introduction

Additive layer manufacturing (ALM) is a bottom-up manufacturing method that can repair or remanufacture high-value components used in a variety of industrial applications, add delicate features to present products, and produce nearly net-shaped complex three-dimensional (3D) products [1,2]. ALM has primarily been applied to quick prototyping, component repair, and reconditioning [3]. Recent developments in ALM have extended its use to include the manufacture of porous materials and functionally graded materials [4]. As a result, the manufacturing industry and manufacturing research community are becoming interested in ALM. Researchers at several national laboratories and prestigious universities are actively making use of the established process’s applicability, while others are employing analytical and numerical techniques to model the process to improve its capabilities [5]. Levy et al. [6] examined ALM processes (both patented and commercialized) for rapid manufacturing and rapid tooling applications. They came to the conclusion that while ALM technology for polymer-based materials has advanced with the commercial availability of numerous rapid prototyping (RP) machines, metallic materials remain a challenge for researchers. They added that one of the key elements that distinguishes different ALM processes is the kind of energy source. The core energy sources utilized in ALM for metallic materials include lasers, electron beams, and electric arcs. For small-scale deposition, lasers and electron beams are chosen because they are more accurate energy sources than electric arcs. However, a noteworthy disadvantage is their low energy conversion efficiency, which results in increased energy consumption for tooling, component manufacturing, and associated applications. The two main problems with these procedures are the use of energy-efficient sources and an increase in deposition rates.

The gas tungsten arc welding (GTAW) procedure was reported to deposit the material and 3D metallic parts were constructed successfully [7]. The fabricated part showed a reliable microstructure throughout and was free of any defects. A 3D structure was formed successfully using micro-sized wire for minuscule deposition using a micro-tungsten inert gas (TIG) welding technique [8]. A 6-axis robotic system in conjunction with laser beam deposition and GTAW has been reported to produce a multi-layered single bead wall [9]. They compared the mechanical characteristics of the deposits made using the two methods. Their findings verify that ALM applications can make use of both laser and GTAW techniques.

One crucial factor in the ALM procedures is the form of filler material. It may take the shape of wire, micron-sized powder, or a mix of the two. Paul et al. [10] have reported that powdered deposition material is perfect for laser-based procedures. On the other hand, because intermittent start and stop cause discontinuity in the deposited material, wire form is recommended for the manufacture of components involving continuous deposition. Additionally, using wire leads to nearly 100% material use efficiency. The adoption of energy-efficient ALM processes to repair and/or remanufacture the problematic dies and molds can greatly increase their performance, life duration, and profitability, according to Jhavar et al. [11], who examined the various failure mechanisms of dies and molds and their repair possibilities. Due to the extremely expensive initial investment and increased running costs, which SMEs cannot afford, the use of high energy beam technologies (such as laser and electron beam) to remanufacture or repair damaged dies and molds is limited. As a result, there is an urgent need to create a low-cost, material-efficient, and energy-efficient method for fixing and remanufacturing the faulty dies and molds.

Therefore, a computational approach is needed to fulfill this need by reducing the trial-and-error experimental cost, money and energy and optimizing the processing parameters to obtain the better deposition rate for providing defect-free products [12,13,14]. CALPHAD and MD simulation approach has been reported for different advanced manufacturing and materials development processes [15]. A CFD simulation model has been developed to investigate the heat transfer phenomena of metal casting process during solidification [16]. A FEM model has been developed to investigate the thermal distribution of micro-plasma wire arc additive manufacturing (MPWAAM) [17]. Numerous industries, including computer science, aviation, healthcare, and manufacturing, have demonstrated the efficacy of machine learning (ML) applications [18,19,20]. Data-driven strategies based on machine learning (ML) technologies have become more widely used in digital manufacturing systems to uncover hidden knowledge and create intricate relationships as data collecting and storage technologies [21,22,23,24]. Wu et al. [25] have reported a review on neural network with SHAP analysis for quality prediction in different complex manufacturing processes. Ackermann et al. [26] have studied the identification of process–structure relation in metal additive manufacturing using ML process. Jain et al. [27] have reported the prediction and validation of alloying element on the mechanical behavior of high entropy alloys produced by manufacturing process to reduce the dependency on the experiments. A comprehensive review of ML approach in ALM process has been reported to identify the research challenges and future opportunities. The most recent uses of machine learning in WAAM, including process monitoring, defect identification, and parameter optimization, are thoroughly reviewed [19]. Recently, Kumar et al. [28] predicted and optimized WAAM process parameters with the help of ML process to enhance the deposition efficiency. In WAAM process, prediction of process instability and CTWD drift estimation has been reported by Oueslati et al. [29]. A review of ML applications on additive manufacturing has been reported by Pazireh et al. [30], in which advanced techniques such as deep learning and other models are included. Sharma et al. [31] have reported the forecasting of process parameters for additive manufacturing process with the help of ML techniques. A Random Forest model with 94% accuracy to predict the layer height and width was reported for SS-316L steel.

While machine learning techniques have been used in additive manufacturing in the past, there are still few thorough comparison studies that use multiple ML models with output-wise interpretability for simultaneous deposition characteristic prediction in MPWAAM wire deposition process. In order to accurately predict and analyze numerous deposition reactions, the current study creates a complete framework that combines seven machine learning models with explainable analysis based on SHAP. Using the basic concepts of energy balance and heat transmission, the current work seeks to create a ML framework to predict the width and height of single-track deposition as a function of MPWAAM wire deposition process parameters and to optimize the processing parameters to obtain the better products.

## 2. Materials and Methods

### 2.1. Data Collection

Any ML process is fundamentally dependent on the data to establish the relationship between input and output parameters. This data is the pillar of the prediction work. The initial step in any ML process is the collection of the dataset. The size of the dataset determines the efficiency of the prediction models and their accuracy level. Therefore, collection of the dataset from simulation studies, experimental results and different literature is very critical. In the present study, the dataset was collected from theoretical calculation and experimental results from the literature.

#### 2.1.1. Theoretical Collection

In the MPWAAM deposition process, the final bead geometry depends on multiple operating parameters such as plasma power (heat input), worktable travel speed, wire diameter, wire feed rate and alignment, stand-off distance, gas flow rates for plasma and shielding, and the thermal properties of the deposited material and the substrate. For the purpose of simplifying the thermal analysis, the following assumptions were introduced [32]:

- The substrate is approximated as a semi-infinite medium maintained initially at ambient temperature *T_i_*.
- The deposited material and substrate are assumed to have identical isotropic and homogeneous characteristics.
- Thermophysical properties are treated as constant, neglecting their dependence on temperature.
- The plasma arc is oriented perpendicular to the substrate surface, and the distance between the torch and substrate remains unchanged.
- The melt pool is assumed to possess a spherical geometry with the pool surface maintained at the substrate melting temperature *T_m_*. Under steady-state conditions, the deposition width corresponds to the pool diameter [33].
- Heat flow within the molten region is assumed one-dimensional along the *y*-axis.
- Heat losses due to radiation and convection are ignored [34].
- The deposition process is symmetric about the *y*-axis, and the geometry remains constant along the torch travel direction (*z*-axis).

The deposition width can be calculated from the following equation [35]:

(1) $$W=2\;R_{m}=2\;[\frac{\eta VI-V_{d}\rho_{d}C_{pd}(T_{md}-\;T_{i})}{17.8\;\rho_{s}\;C_{ps}\left(T_{ms}-\;T_{i}\right)\;\sqrt{\alpha_{s}\;v}\;[erfc\left(1\right)-\;\frac{1}{exp\sqrt{\pi }}+\;\frac{1}{\sqrt{\pi }}]}\;{]}^{2/3}$$

Here, *η* represents the thermal efficiency of the micro-plasma transferred arc (%). V denotes the DC voltage applied to the plasma torch (V), while I indicates the DC current supplied to the torch (A). *V*_*d*_ is the volumetric deposition rate (mm^3^/s), and *ρ*_*d*_ is the density of the deposited material (kg/mm^3^). *C_pd_* refers to the specific heat capacity of the deposition material (J/kg·K), *T_md_* is the melting temperature of the deposition material (K), and T_i_ represents the ambient temperature (K). Furthermore, *ρ_s_* denotes the density of the substrate material (kg/mm^3^), *C_ps_* is the modified specific heat of the substrate (J/kg·K), and *T_ms_* is the melting temperature of the substrate material (K). The thermal diffusivity of the substrate is represented by α_s_ (mm^2^/min), while v indicates the travel speed of the worktable (mm/min).

The deposition height can be calculated from the following equation:

(2) $$h\;=\;\frac{4\;\left(1-D\right)\;V_{d}}{\pi vw}$$

where *D* is the dilution percentage, *V_d_* denotes the volumetric deposition rate (mm^3^/s), *v* represents the movement speed of the worktable (mm/s), and w indicates the deposition width (mm), evaluated according to Equation (1).

The energy per unit worktable speed in J/mm and volumetric deposition rate per worktable speed in mm^3^/mm can be calculated from the following equations:

(3) $$E_{l}\;=\;\frac{60\;P}{V}$$

(4) $$V_{l}=\frac{A_{w}\;f}{v}$$

With the help of these 4 equations and varying the processing parameters of plasma power, worktable speed and wire feed rate, 2070 datasets were generated for the prediction and validation work in the present study. The range of the varying parameters was selected from the machine specifications as shown in Table 1.

#### 2.1.2. Data Collection from the Literature

With these theoretical values, some experimental values are also collected to balance the dataset and to enhance the reliability of the dataset. In total, 42 experimental datasets were collected from the published literature [32,35]. The dataset contains measured outputs like bead width and height as well as process characteristics like laser power, travel speed, and wire feed rate. This guarantees that the data utilized in this investigation is fully traceable and reproducible. The physical consistency of the theoretically created dataset is evaluated using these experimental data points.

Therefore, 2112 datasets were prepared for the present study to train the different models and to predict the different output parameters to reduce the experimental dependency and to predict the behavior in a better way. The collected dataset was complete and free of missing values. Moreover, all input features were numerical variables; therefore, no missing value imputation or categorical feature encoding was required prior to model training.

The dataset used in the present study was generated using a combination of validated theoretical relationships and experimentally measured data related to deposition characteristics. Most of the output parameters were calculated using theoretical formulations, while part of the dataset was obtained from experimental observations. These formulas are based on known relationships that control material deposition, heat input, and bead shape in MPWAAM wire deposition. It is important to note that these theoretical relationships have previously been validated through experimental studies conducted by Nikam et al. [32] Therefore, the generated dataset maintains strong physical relevance and experimental reliability. The remaining 42 data points represent actual process activity and match experimentally measured values.

### 2.2. Development of ML Algorithms

After preparing the dataset, selection of ML algorithm is the most important step. In the present study, seven different ML algorithms such as Random Forest (RF), Gradient Boosting (GB), XGBoost, Extra Tree (ET), Support Vector Regressor (SVR), Elastic Net (EN) and LGBM were chosen based on their strengths and weaknesses in handling the complex dataset. In order to provide a thorough comparison of prediction performance, these models were chosen to reflect both ensemble-based approaches and traditional machine learning techniques.

To optimize these algorithms for better results, hyperparameter tuning approach was used with the help of a five-fold cross-validation technique, which allows us to perform iterative adjustment of different hyperparameters through repeated training and testing by improving their prediction skills.

The data was normalized using the minmax scalar before it was fed into the models. After successful development of these models, predicted values were denormalized to return them to the original scale. The collected dataset was divided into three parts in the ratio of 60:20:20 used for training, testing and validation, respectively, by ensuring robust model evaluation and minimizing the risk of overfitting with the help of random splitting strategy. This ratio was selected to ensure a balanced trade-off between model training, hyperparameter tuning, and unbiased performance evaluation. To guarantee reproducibility, a fixed random seed was employed. The dataset was analyzed before splitting to ensure that the output responses and input parameter distributions (laser power, travel speed, and wire feed rate) were fairly uniform. Each subset thus maintains the general statistical properties of the entire dataset. This training set was used to build and tune the different ML models to optimize in a better way. The testing set was used as an intermediate checkpoint during the optimization work. After optimizing the models, the validation set was used to evaluate the performance of the finalized model because this dataset was kept isolated from the model optimization process.

Careful checks were carried out to prevent data leaking in order to guarantee the veracity of the results. Before the model was trained, the dataset was divided, and neither the validation nor the test sets were utilized in the model’s development.

**Figure 1:**
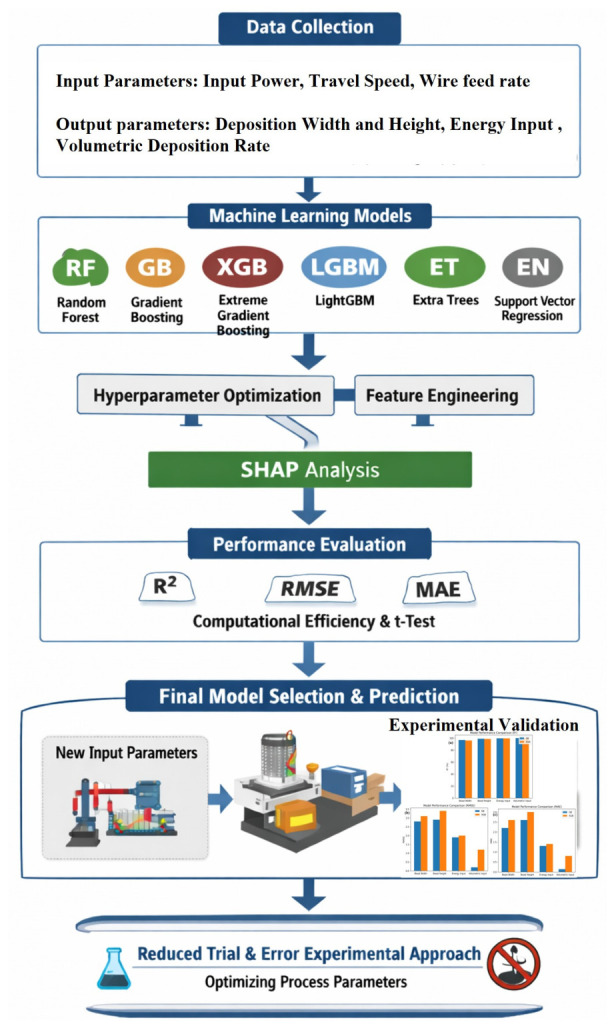
A complete methodology with the different steps used in the present study.

This whole procedure was conducted on a workstation with an Intel Core i7 processor, 128 GB RAM and NVIDIA GeForce RTX 3070 GPU. The whole analysis was carried out using the Python library “scikit-learn 1.3.1”. The complete methodology with the different steps is shown in Figure 1.

### 2.3. Feature Engineering

Feature engineering is also an important step to select the most relevant features by seeing their correlation with output parameters to ensure that only significant parameters are included to develop the different ML models. Pearson and Spearman correlation analyses were used to examine the connections between processing parameters and the goal variable. While Spearman correlation is resilient to nonlinear dependencies and analyzes monotonic relationships based on rank-order, Pearson correlation evaluates linear relationships.

Using the best machine learning model, SHAP (SHapley Additive exPlanations) analysis was carried out to understand the impact of processing parameters on the deposition behavior. To measure the individual and combined effects of the descriptors on the anticipated deposition behavior, SHAP dependence graphs were generated. The magnitude and direction of each descriptor’s contribution to the anticipated deposition behavior attribute can be seen using the SHAP dependence charts.

### 2.4. Model Performance Evaluation

Three widely used regression measures were employed to assess the ML models’ prediction performance. While RMSE and MAE quantify the size of prediction errors, the *R*^2^ metric quantifies the percentage of variance in the target variable explained by the model. Better predictive performance is shown by higher *R*^2^ values and lower RMSE and MAE values. Model fitting and generalization ability were evaluated across training, validation, and testing datasets. The validation performance was used for model comparison instead of training and testing performance for reliability.

To evaluate the performance in a better way, computational efficiency was assessed. Two important indicators were taken for it. The amount of time which is desirable to train the model and the memory requirements. The scalability and computational cost of used ML models can be confirmed with the help of these two metrics.

A paired *t*-test was performed on the prediction errors in order to statistically inspect the variations in predictive performance. The *t*-test establishes whether a difference in mean prediction errors between two models is the result of random variation or is statistically significant. This significance was evaluated using a *p*-value threshold of 0.05. The performance difference between two models is deemed statistically significant if the *p*-value is less than 0.05.

The final model selection was based on a comprehensive evaluation process that took into account the statistical significance of performance fluctuations, computational effectiveness, and the results based on test and validation datasets.

## 3. Results

### 3.1. Deviation Analysis Between Experimental and Theoretical Calculated Values

**Figure 2:**
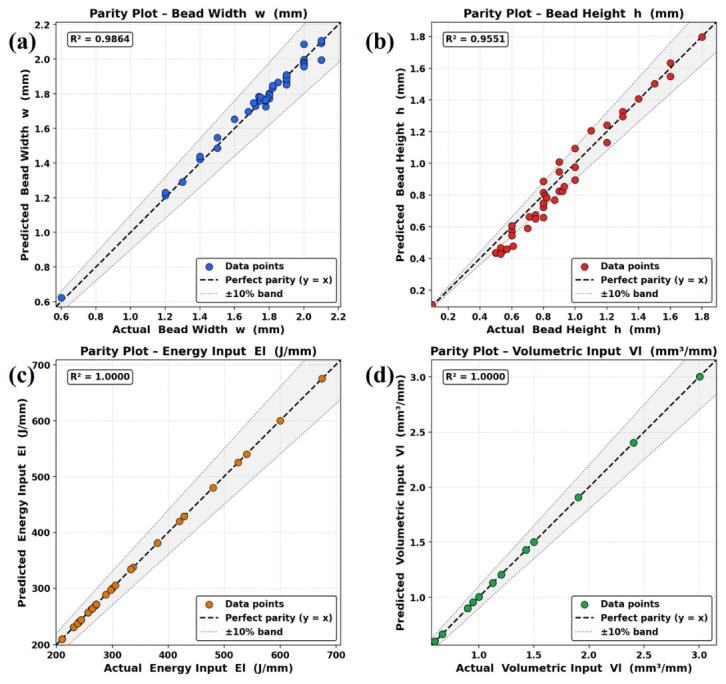
Parity plots comparing theoretical predictions and experimentally reported values for (**a**) bead width, (**b**) bead height, (**c**) energy input, and (**d**) volumetric input. The dashed line represents ideal agreement (y = x), while the dotted lines indicate the ±10% error band.

**Figure 3:**
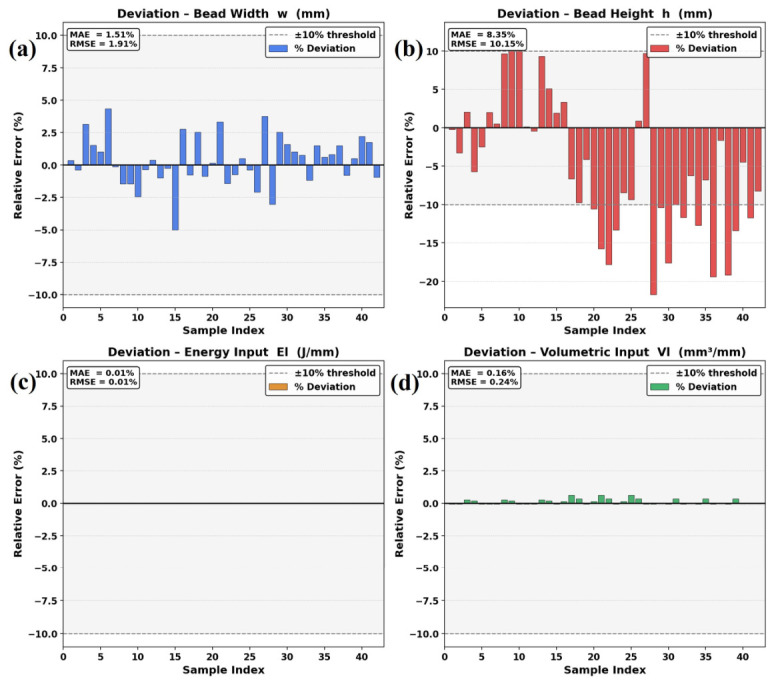
Relative deviation plots between theoretical and experimental values for (**a**) bead width, (**b**) bead height, (**c**) energy input, and (**d**) volumetric input across the 42 experimental samples. The dashed lines represent the ±10% deviation threshold.

As seen in Figure 2 and Figure 3, parity plots and deviation analyses were carried out for the 42 experimental samples in order to evaluate the concordance between theoretical predictions and experimentally recorded values. The parity plots show that there is good consistency between theoretical and experimental results, with the majority of data points being closely distributed around the ideal agreement line (y = x). Strong agreement was confirmed by the obtained R^2^ values of 0.9864 for bead width and 0.9551 for bead height. Since these parameters are directly controlled by deterministic process equations, energy input and volumetric input showed almost perfect agreement. The theoretical formulations’ dependability is further validated by the deviation analysis. With MAE and RMSE values of 1.51% and 1.91%, respectively, bead width showed comparatively little variances. Bead height showed somewhat greater variances (MAE = 8.35%, RMSE = 10.15%), which might be explained by the thermal model’s oversimplified assumptions and the experimental variability documented in the literature. Crucially, there was no discernible systematic trend of overprediction or underprediction.

### 3.2. ML Performance

#### 3.2.1. Training, Validation and Testing Performance

**Figure 4:**
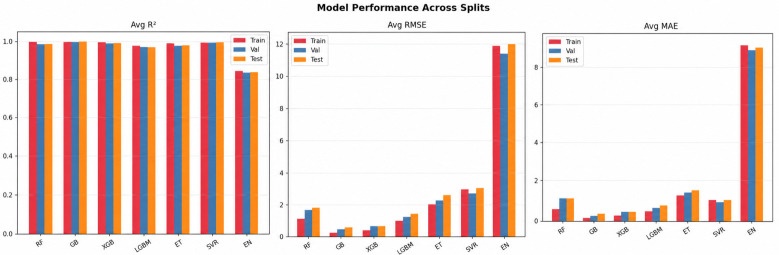
Training, Testing and Validation Performance of all used ML models.

Using three statistical performance metrics across the training, validation, and testing datasets, Figure 4 compares the predictive performance of the machine learning models, including all seven ML algorithms. It is clear from Figure 2 that the tree-based ensemble models (GB, XGB, LGBM, and ET) generally show better prediction performance compared to the others. These models show good agreement between the expected and actual values, achieving very high R^2^ with low RMSE and MAE values. The GB and XGB models perform exceptionally well, retaining high accuracy across training, validation, and testing datasets, indicating remarkable generalization capabilities without severe overfitting.

In comparison to boosting-based models, the RF model has somewhat higher RMSE and MAE values but likewise determines a comparatively strong predictive potential. The ET and LGBM models show similar performance with moderate error values, signifying that they are appropriate for capturing nonlinear interactions. The SVR model has low predictive performance with higher error metrics, indicating a limited capacity to capture the intricate nonlinear relationships among processing factors. This suggests that the nonlinear behavior of the process parameters and reactions is more difficult to model using linear models.

The dependability of the trained models for predicting the deposition features is further confirmed by the strong agreement between training, validation, and testing metrics for the majority of ensemble models, which shows good model stability and little overfitting. The Gradient Boosting (GB) model demonstrated the greatest overall predictive performance across all the algorithms studied, while the XGBoost (XGB) model came in second. Both models achieved reduced RMSE and MAE and higher *R*^2^ values.

Across training, validation, and testing datasets, the Gradient Boosting model shows extremely high *R*^2^ values that are close to unity. Although such performance is rare in complicated physical systems, it can be explained by the dataset’s structured character, which is primarily generated from theoretical formulations based on physics. The model is able to learn near-deterministic patterns because these formulations enforce tight correlations between process parameters and output responses. Therefore, the high predictive accuracy reflects the consistency of the underlying physical relationships rather than unintended information leakage.

#### 3.2.2. Computational Efficiency of ML Models

**Figure 5:**
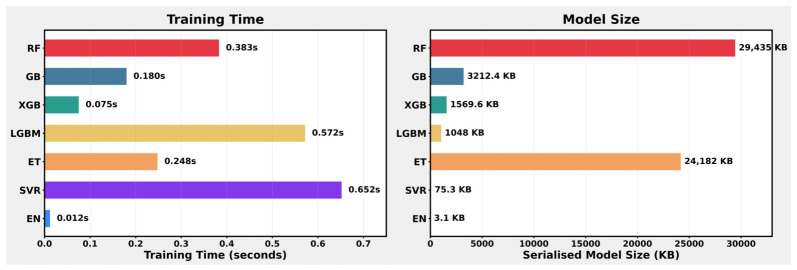
Performance of ML models based on computational efficiency.

The computational efficiency of all used ML models is shown in Figure 5 in the form of training time and model size. Figure 5 shows that GB had the fastest training time (0.18 s) of all the used models while retaining robust predictive performance, representing its computational efficiency for large-scale applications. Furthermore, the model size was 3212.4 KB for the GB model, suggesting a reasonable memory demand for model deployment and storage. Although XGB and EN also had relatively fast training times, they had less prediction performance.

On the other hand, ensemble models like RF and ET produced higher model sizes and longer training times. They have better predicted accuracy, but these models may not be as effective in real-time applications due to their increased processing cost. With a minimum training time (0.075 s) and a relatively small model size (~1569.6 KB), XGBoost showed a balanced trade-off between computational cost and predictive performance. These results show that, in comparison to previous ensemble techniques, GB and XGB offer better computational efficiency, even while ensemble models give superior predictive performance.

Overall, the computational analysis supports the Gradient Boosting model’s applicability for effective deposition characteristic prediction by confirming that it not only offers improved predictive performance but also maintains acceptable training time and model size.

#### 3.2.3. Statistical Significance Analysis

The prediction errors of the assessed algorithms were subjected to a paired *t*-test in order to compare the prediction performance of the GB model with other ML models, in order to further assess the dependability of the top-performing model. The sample size (n) and degrees of freedom (df) were specifically included for both cross-validation and independent test comparisons in order to enhance the statistical interpretation of the paired *t*-test analysis. The sample size (n) corresponds to the number of data points in the test set, and the degrees of freedom were calculated as

df=n−1

For the cross-validation (CV) analysis, paired *t*-tests were performed using the five-fold CV scores (n = 5, df = 4). The prediction errors from 423 test samples (n = 423, df = 422) were used to perform the paired *t*-test for the independent test-set analysis. Stronger statistical reliability for assessing variations between model predictions is provided by the test comparison’s much larger sample size.

The results of *t*-test are shown in Table 2. The statistical analysis reveals that the differences between the GB model and the other models are statistically significant because the t-statistic values are positive and relatively big, and all comparisons’ *p*-values are below the significance threshold of 0.05, indicating that their prediction-error distributions were significantly different from that of the GB model. Specifically, the comparison with SVR and EN shows extremely high t-statistic values, indicating a significant performance gap between these models and the GB model. These findings support the GB model’s selection as the best model for additional study and interpretation by demonstrating that it performs noticeably better than the other machine learning algorithms in terms of predicted accuracy.

### 3.3. Model Selection and Implications

Based on the collective assessment of predictive performance, computational efficiency, and statistical significance, the ensemble-based ML models showed superior capacity in forecasting the processing parameters. With the highest R^2^ values and the lowest RMSE and MAE, the GB model demonstrated the best overall performance among the used algorithms, indicating strong generalization ability. The second-best performer was the XGB model, which again produced very precise predictions with consistent performance across data splits. Additionally, the computational analysis showed that the GB model retains a moderate model size and an efficient training time, making it appropriate for real-world application. The performance differences between the GB model and other algorithms are statistically significant (*p* < 0.05) according to a paired *t*-test statistical comparison. Overall, these findings show that ensemble boosting techniques such as GB and XGB are very successful at modeling intricate nonlinear relationships in datasets related to materials processing, offering better robustness and dependable predictive capability when compared to traditional ML models.

### 3.4. Prediction Interval and Uncertainty Analysis

**Figure 6:**
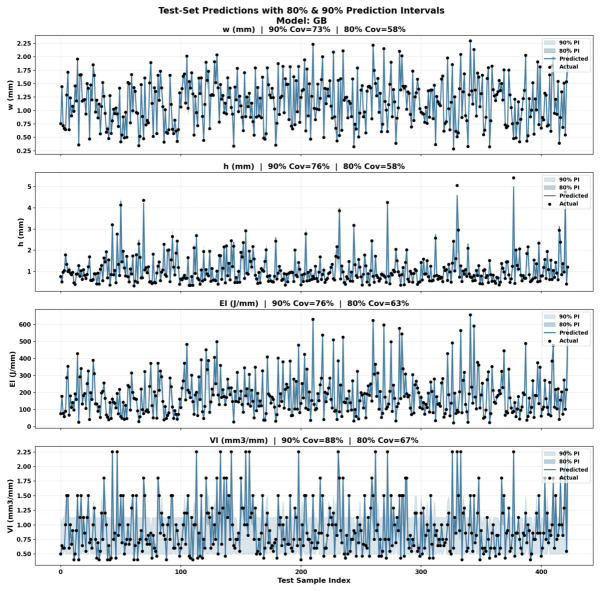
Prediction interval analysis for the best-performing GB model showing actual and predicted values along with 80% and 90% prediction intervals for w, h, EI, and VI. The coverage percentages indicate the proportion of experimental data captured within the corresponding prediction intervals.

As seen in Figure 6, prediction interval (PI) analysis was carried out for the top-performing Gradient Boosting (GB) model in order to further assess the robustness and dependability of the created ML framework. Bead width (w), bead height (h), energy input (EI), and volume indicator (VI) were among the output variables for which the 80% and 90% prediction ranges were calculated.

The findings show that the GB model has strong predictive consistency since the majority of the experimental observations fit within the anticipated uncertainty bounds. (VI) showed the highest interval coverage among the outputs taken into consideration, but (w) and (h) showed significantly lesser coverage, indicating substantially higher variability in these responses. Overall, the prediction interval analysis verifies that the suggested machine learning architecture enables appropriate uncertainty estimation for real-world process optimization applications in addition to precise point predictions.

For additional examination, additional uncertainty distribution and prediction interval comparison graphs for several machine learning models are included in the Supplementary Materials.

### 3.5. SHAP Analysis

**Figure 7:**
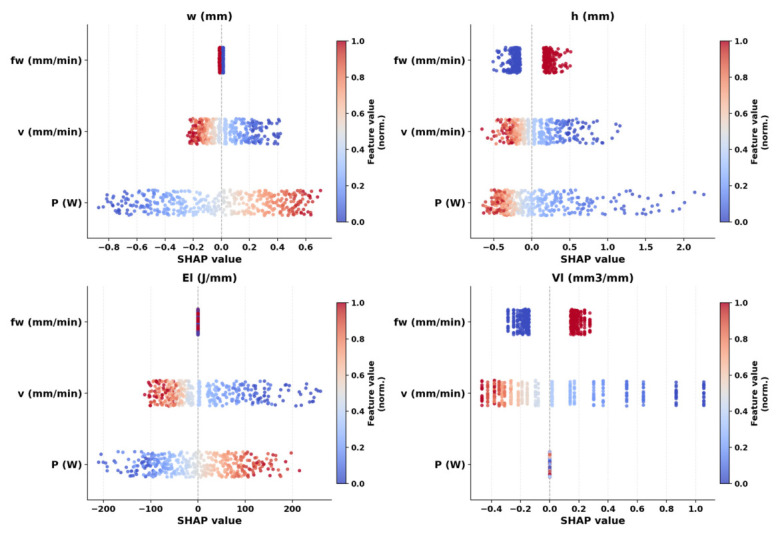
SHAP summary analysis for the best-performing GB model.

The best-performing Gradient Boosting (GB) model was used to conduct independent SHAP analyses for each output since SHAP feature relevance varies according to the target response variable. While the SHAP dependence charts in Figure 5 offer additional insight into the interaction behavior and impact of input features on each output variable, the SHAP summary plots in Figure 7 demonstrate the distribution and contribution of process parameters for specific responses.

#### 3.5.1. SHAP Summary Analysis of the Best GB Model

The SHAP summary graphs for the GB model are shown in Figure 7, emphasizing the directional influence and relative contribution of the input parameters such as travel speed (v), wire feed rate (fw) and laser power (P) on the expected responses. Figure 7 confirms the need for distinct SHAP interpretation for each output variable by showing that the contribution and influence of process factors vary dramatically depending on the target response. Laser power has the greatest spread in SHAP values for bead width (w), suggesting that it is the most significant factor. While lower power values (blue points) decrease the anticipated width, higher power values (red points) primarily contribute favorably to the bead width prediction. Because there is less heat input per unit length at greater travel speeds, bead width tends to shrink. Wire feed rate has a relatively small impact on breadth.

Both laser power and travel speed are important factors when it comes to bead height (h). While higher wire feed rates result in positive SHAP contributions, suggesting that higher material flow encourages vertical deposition, higher travel speeds often have a detrimental impact on the bead height. It is evident that laser power and travel speed have a major impact on energy input (EI). While higher travel speeds lower the energy input because of a shorter interaction period between the heat source and the substrate, higher power values significantly contribute to positive SHAP values, raising EI.

The most significant factor affecting volumetric input (VI) is wire feed rate; greater feed rates result in higher volumetric input. Higher travel speeds, on the other hand, have a negative impact, meaning that at faster scanning rates, there is less deposited volume per unit length.

Overall, the SHAP summary plots show that travel speed influences both the thermal and geometric properties of the deposited bead, wire feed rate expressively affects VI, and laser power primarily controls w and EI. These patterns align with the basic physics of additive manufacturing.

#### 3.5.2. Effect of Each Input Parameter on Output Parameter Using Top-Performing Model

**Figure 8:**
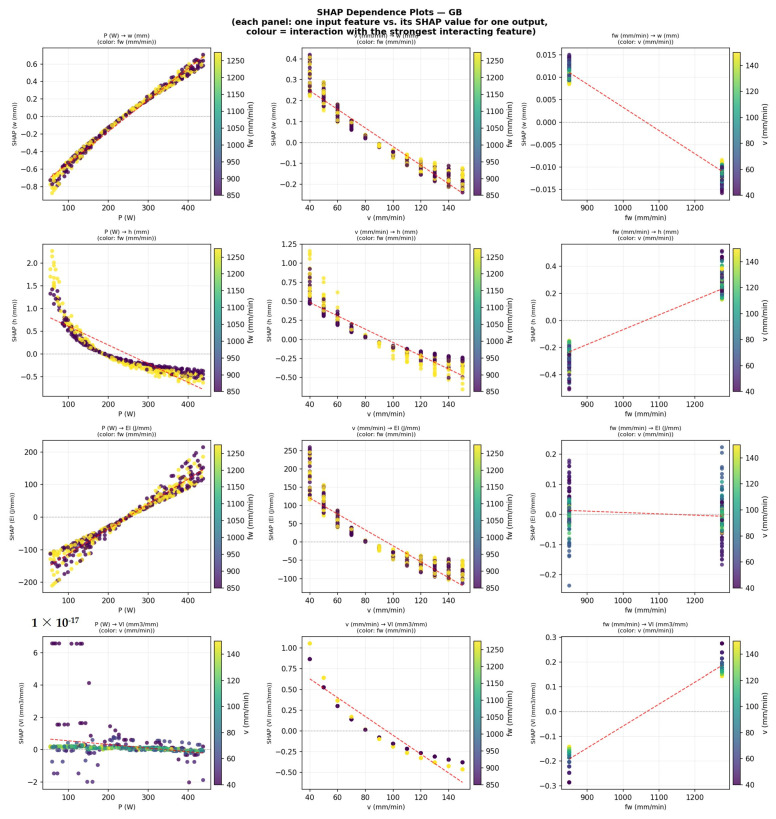
SHAP dependence plots using the best-performing GB model.

The SHAP dependence plots for the Gradient Boosting model are shown in Figure 8, which shows how the process parameters such as laser power, travel speed and wire feed rate affect the anticipated responses, which include bead width, bead height, energy input, and volumetric input. According to the SHAP study, there is a significant positive correlation between laser power (P) and bead width. The SHAP value of width grows almost linearly as the power increases from around 80 W to 420 W, suggesting that higher power greatly contributes to wider deposited beads. On the other hand, high power may encourage lateral spreading of the molten pool rather than vertical growth, as indicated by the nonlinear declining trend of the influence of power on bead height (h).

The weakening SHAP values with growing travel speed indicate a negative association between the travel speed and bead geometry. This suggests that the potential heat input is decreased at larger speeds, leading to lower bead dimensions. The fw parameter has a sensibly small impact on bead width but a moderate effect on bead height. Higher feed rates typically result in more material being deposited, which raises the height of the bead. The probable physical connection between these process variables is confirmed for the computed parameters, where EI increases intensely with increasing power and declines with increasing travel speed. Similar to this, travel speed and wire feed rate have a significant impact on volumetric input (VI), with greater feed rates increasing deposited volume and higher travel speeds decreasing it. The charts’ color gradients also show how variables interact with one another. Bead width and energy input are particularly influenced by the interplay between power and wire feed rate, indicating that coordinated modification of these parameters is necessary to achieve optimal deposition characteristics.

Overall, the SHAP dependence analysis confirms the physical understanding of the wire arc additive manufacturing process by confirming that laser power and travel speed are the most important factors, whereas wire feed rate primarily influences the material deposition rate and bead height.

### 3.6. Prediction and Experimental Validation

**Figure 9:**
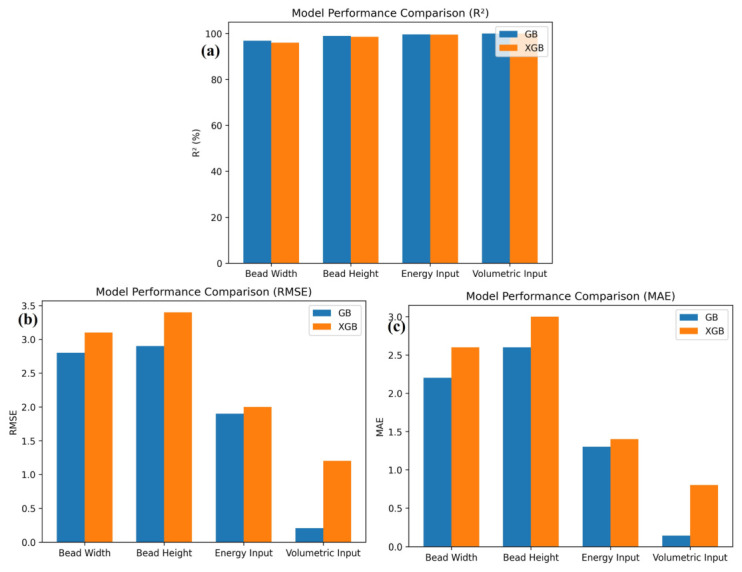
New prediction performance using GB and XGB models, showing (**a**) R^2^ (**b**) RMSE and (**c**) MAE (All in %).

To get more reliable results, a set of input parameters by varying the laser power, work travel speed and wire feed rate was selected by showing their effect on the output parameters obtained from SHAP analysis. The two top-performing ML models, GB and XGB, were used to predict all four output parameters. The predicted output was then compared with the experimental results obtained from the experimental study reported in the previous literature. The comparison of predicted and experimental output values is shown in Table 3 and Table 4. The performance of these two models is shown in Figure 9. The comparison showed that there was good agreement between the experimental and predicted data, suggesting that the generated models can accurately capture the underlying relationships between deposition properties and process parameters. The ensemble models’ resilience and capacity for generalization are further demonstrated by the little discrepancy between reported experimental values and anticipated values. This validation validates the suggested data-driven framework’s potential for accurate deposition behavior prediction in advanced additive manufacturing applications.

## 4. Discussion

The current findings demonstrate how well ensemble machine learning techniques can represent intricate interactions between deposition responses and process parameters in advanced additive manufacturing. Specifically, the Gradient Boosting and Extreme Gradient Boosting models’ improved performance shows how well boosting algorithms capture nonlinear interactions in materials processing datasets. The robustness and dependability of the chosen model for predictive applications are further supported by the statistical significance analysis.

Furthermore, the SHAP-based interpretability analysis offers significant insights into the relative impact of important process parameters, showing that wire feed rate primarily regulates volumetric deposition behavior, while laser power and travel speed primarily control energy input and bead geometry. The robustness and practical applicability of the ensemble machine learning models for predicting deposition characteristics are further confirmed by the close agreement between the predicted results and previously published experimental data. These results show how explainable machine learning can improve process comprehension and are compatible with the underlying process physics. To further enhance prediction accuracy and facilitate real-time process optimization in advanced additive manufacturing systems, future research may concentrate on adding more process variables and larger experimental datasets.

It is important to note that the strong predictive performance of the models, particularly Gradient Boosting, may partly reflect the structured nature of the theoretically generated dataset. Since a significant portion of the data follows predefined physical relationships, the models may inherently learn these trends. Nevertheless, the SHAP-based feature importance remains physically consistent, indicating that the model captures meaningful process–property relationships rather than purely statistical correlations.

It is important to know that the underlying dataset is impacted by oversimplified physical assumptions, even when the model predictions exhibit strong agreement with anticipated trends. As a result, rather than capturing higher-order nonlinearities resulting from melt pool circulation, radiation losses, and temperature-dependent characteristics, the machine learning models might mainly capture first-order process interactions.

## 5. Conclusions

Based on the process parameters of laser power (P), travel speed (v), and wire feed rate (fw), several machine learning models were created and assessed in this study to predict important deposition characteristics, such as bead width (w), bead height (h), energy input (EI), and volumetric input (VI). Using R^2^, RMSE, and MAE metrics across training, validation, and testing datasets, the predictive performance of seven models used, including RF, GB, XGB, LGBM, ET, SVR, and EN, was methodically evaluated.

The results show that when it comes to capturing the nonlinear interactions between processing parameters and deposition responses, ensemble-based models perform better than traditional ML algorithms. The GB model outperformed the other models in terms of overall predictive performance, followed by the XGB model, both of which demonstrated great generalization capabilities and high accuracy. The GB model offers a good balance between prediction accuracy, training time, and model size. Furthermore, a paired *t*-test validated the robustness of the chosen model by confirming that the performance differences between the GB model and other models are statistically significant (*p* < 0.05). In addition, prediction interval analysis demonstrated the reliability and robustness of the GB model by quantifying the uncertainty associated with the predicted deposition responses.

The nonlinear interactions between these parameters and their contributions to the deposition features were further brought to light by the SHAP dependence analysis. The interpretation results showed that while wire feed rate is the primary factor influencing volumetric deposition behavior, laser power and travel speed are the most significant factors influencing energy input and bead geometry. The efficacy of the suggested data-driven approach for accurate prediction of deposition properties in advanced additive manufacturing is demonstrated by the successful validation of the GB and XGB model predictions against previously published experimental results.

Overall, this study displays that explainable ML methods in conjunction with ensemble boosting models offer a well-organized framework for anticipating and deciphering intricate process–structure connections in advanced manufacturing processes. The suggested method supports the formation of more effective and dependable production strategies by providing insightful information for process optimization and parameter selection. Although the present dataset is supported by experimentally validated theoretical relationships and available experimental data, future work may include additional independent experiments for further external validation and industrial implementation of the proposed ML framework.

## 6. Limitations and Future Work

Regarding the benefits of the current strategy, it is important to recognize its limitations. There are only a few experimental examples in the collection, which is mostly made up of theoretically produced data (~98%). The model may still be skewed toward idealized process behavior and may not adequately account for experimental uncertainties, noise, and unmodeled phenomena, despite the theoretical formulations’ physical validation and foundation in earlier experimental investigations. Future research will concentrate on testing the suggested framework under various processing settings, adding real-time process variability, and growing the experimental dataset to enhance model generalization.

Under circumstances where heat transport processes and material behavior differ significantly from the idealized assumptions, these simplifying assumptions may limit the generality of the suggested model.

Under optimal circumstances, the model may achieve very high predicted accuracy because the theoretically created dataset is somewhat deterministic. However, when applied to solely experimental datasets, the model performance may be somewhat worse since real-world processes incorporate extra sources of uncertainty.

## Figures and Tables

**Table 1:** Ranges of the input parameters used in the present study.

S. No.	Parameter Name	Range of the Parameter
1	Plasma Power (W)	55–450
2	Travel Speed of worktable (mm/min)	40–160
3	Wire feed rate (mm/min)	850–1700

**Table 2:** The performance of statistical significance analysis of used ML models.

Model	CV				Test			
	n	df	t-Stat	*p* Value	n	df	t-Stat	*p* Value
RF	5	4	4.8947	0.0081	423	422	−6.005	0
XGB	5	4	6.1678	0.0035	423	422	−0.762	0.4463
LGBM	5	4	12.4018	0.0002	423	422	−3.507	0.0005
ET	5	4	6.3532	0.0031	423	422	−4.726	0
SVR	5	4	21.2308	0	423	422	−3.643	0.0003
EN	5	4	53.5412	0	423	422	−8.543	0

**Table 3:** Comparison of predicted and experimental output values using GB model.

S. No.	P (W)	v (mm/min)	fw (mm/min)	w (mm)		h (mm)		EI (J/mm)		VI (mm^3^/mm)	
				Pred.	Exp.	Pred.	Exp.	Pred.	Exp.	Pred.	Exp.
1	350	40	1275	1.642	1.6	1.368	1.4	525.496	525	2.253	2.2482
2	350	50	1275	1.503	1.5	1.281	1.3	418.490	420	1.802	1.8028
3	350	80	1275	1.237	1.2	0.974	1	263.688	262.5	1.126	1.1241
4	350	40	850	1.566	1.5	1.176	1.2	525.487	525	1.502	1.4988
5	350	50	850	1.326	1.3	0.957	1	418.480	420	1.201	1.2019
6	350	80	850	1.456	1.4	0.683	0.7	263.690	262.5	0.751	0.7494
7	400	40	1275	1.953	1.9	1.44	1.4	596.504	600	2.253	2.2482
8	400	50	1275	1.869	1.8	1.171	1.2	477.717	480	1.802	1.8028
9	400	80	1275	1.89	1.9	0.866	0.9	298.894	300	1.126	1.1241
10	400	40	850	1.914	1.8	0.882	0.9	596.541	600	1.502	1.4988
11	400	50	850	1.741	1.7	0.790	0.8	477.754	480	1.201	1.2019
12	400	80	850	1.807	1.8	0.604	0.6	298.936	300	0.751	0.7494
13	450	40	1275	2.220	2.2	1.259	1.3	656.597	675	2.253	2.2482
14	450	50	1275	2.107	2.1	1.127	1.1	525.190	540	1.802	1.8028
15	450	80	1275	1.702	1.7	0.706	0.7	328.310	337.5	1.126	1.1241
16	450	40	850	1.933	1.9	0.8522	0.9	656.66	675	1.502	1.4988
17	450	50	850	2.109	2.1	0.764	0.8	525.233	540	1.201	1.2019
18	450	80	850	1.920	2	0.412	0.4	328.346	337.5	0.751	0.7494

**Table 4:** Comparison of predicted and experimental output values using XGB model.

S. No.	P (W)	v (mm/min)	fw (mm/min)	w (mm)		h (mm)		EI (J/mm)		VI (mm^3^/mm)	
				Pred.	Exp.	Pred.	Exp.	Pred.	Exp.	Pred.	Exp.
1	350	40	1275	1.642	1.6	1.389	1.4	522.032	525	2.250	2.2482
2	350	50	1275	1.569	1.5	1.283	1.3	419.128	420	1.802	1.8028
3	350	80	1275	1.239	1.2	0.940	1	263.202	262.5	1.125	1.1241
4	350	40	850	1.566	1.5	1.159	1.2	522.234	525	1.507	1.4988
5	350	50	850	1.319	1.3	0.949	1	419.351	420	1.203	1.2019
6	350	80	850	1.459	1.4	0.6743	0.7	263.414	262.5	0.751	0.7494
7	400	40	1275	2.025	1.9	1.3704	1.4	597.505	600	2.242	2.2482
8	400	50	1275	1.827	1.8	1.1863	1.2	477.885	480	1.796	1.8028
9	400	80	1275	1.888	1.9	0.8765	0.9	299.191	300	1.117	1.1241
10	400	40	850	1.848	1.8	0.87	0.9	597.153	600	1.505	1.4988
11	400	50	850	1.762	1.7	0.7686	0.8	477.555	480	1.203	1.2019
12	400	80	850	1.807	1.8	0.5705	0.6	298.849	300	0.749	0.7494
13	450	40	1275	2.212	2.2	1.286	1.3	653.825	675	2.199	2.2482
14	450	50	1275	2.097	2.1	1.0652	1.1	524.285	540	1.767	1.8028
15	450	80	1275	1.759	1.7	0.7218	0.7	328.497	337.5	1.121	1.1241
16	450	40	850	1.964	1.9	0.8389	0.9	653.709	675	1.466	1.4988
17	450	50	850	2.109	2.1	0.7799	0.8	524.190	540	1.178	1.2019
18	450	80	850	1.916	2	0.418	0.4	328.391	337.5	0.756	0.7494

## Data Availability

Data will be available on reasonable request. GitHub link: https://github.com/sjain-ml/Additive-Manufacturing-Data (accessed on 25 May 2026).

## Supplementary Materials

The following supporting information can be downloaded at: https://www.mdpi.com/article/10.3390/mi17060663/s1. Figure S1. Predicted vs. Actual value for Test dataset. Figure S2. Prediction Interval (80% PI) for Test dataset. Figure S3. Prediction Interval (90% PI) for Test dataset. Figure S4. PI coverage rate and Mean width for 80% PI and 90% PI using test dataset. Figure S5. Predictive Uncertainty Distribution using test dataset. Figure S6. Uncertainty vs. Absolute Residual for 90% PI using test dataset. Figure S7. R2 values for each output using the test dataset for all used ML models.
