# Supplementary material for: Explainable Ensemble Machine Learning for Predicting Deposition Characteristics in Advanced Additive Manufacturing
Source: Micromachines (Basel). 2026 May 27;17(6):663. doi: 10.3390/mi17060663 (PMC13303639; doi:10.3390/mi17060663)
Supplement: Supplementary file 1 [file micromachines-17-00663-s001.zip › micromachines-4272445-supplementary.pdf]

Supplementary Materials

Explainable Ensemble Machine Learning for Predicting Deposition Characteristics in Advanced Additive Manufacturing

Sandeep Jain [1,*] and Pradyumn Kumar Arya [2]

[1] School of Materials Science and Engineering, Yeungnam University, Gyeongsan 38541, Republic of Korea
[2] Department of Mechanical Engineering, Indian Institute of Technology Delhi, Hauz Khas, New Delhi 110016, India; pradyumn.mec@gmail.com
* Correspondence: sandeepmbm20@gmail.com

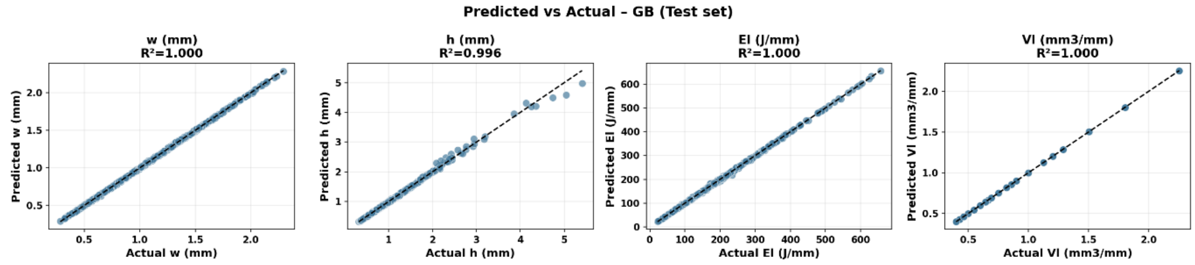

Figure S1. Predicted vs. Actual value for Test dataset.

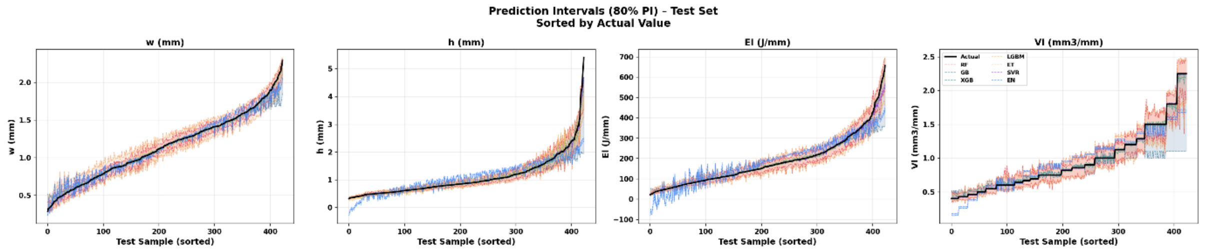

Figure S2. Prediction Interval (80% PI) for Test dataset.

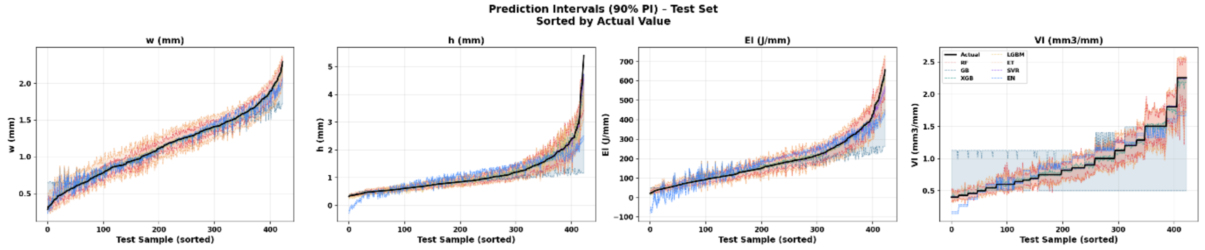

Figure S3. Prediction Interval (90% PI) for Test dataset.

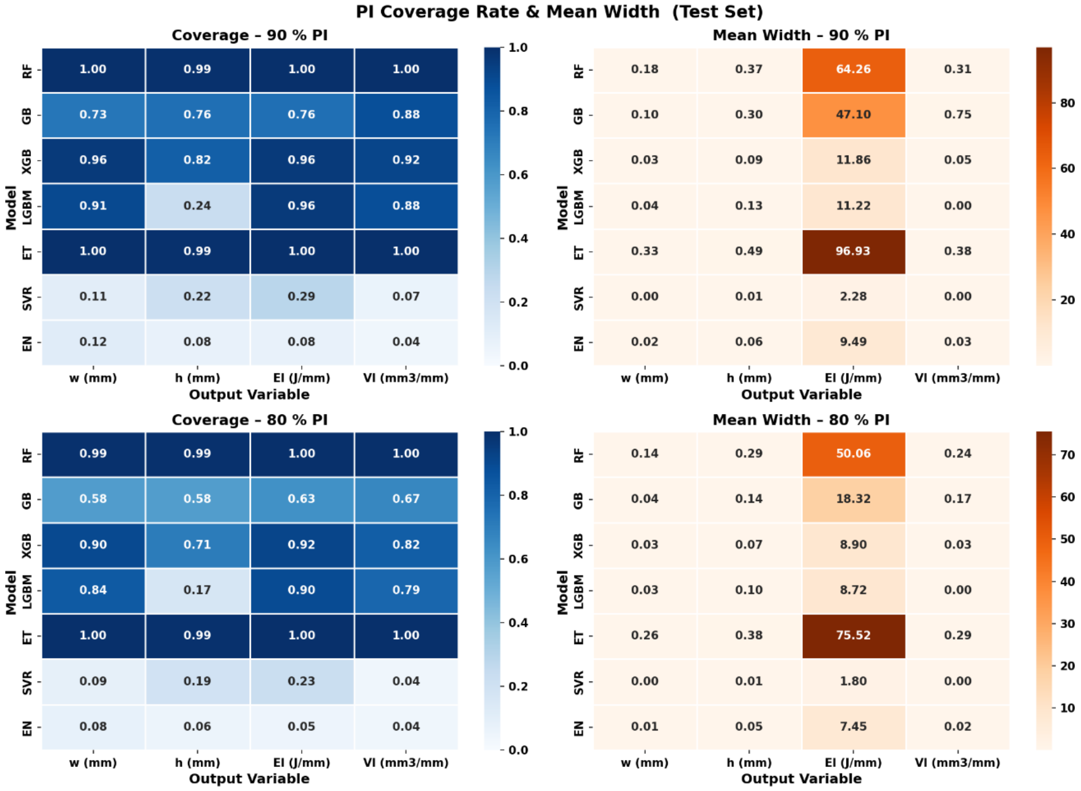

Figure S4. PI coverage rate and Mean width for 80% PI and 90% PI using test dataset.

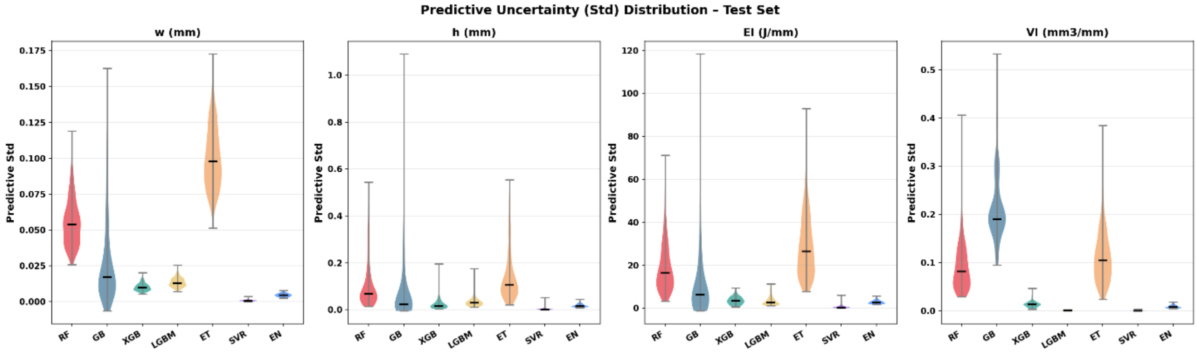

Figure S5. Predictive Uncertainty Distribution using test dataset.

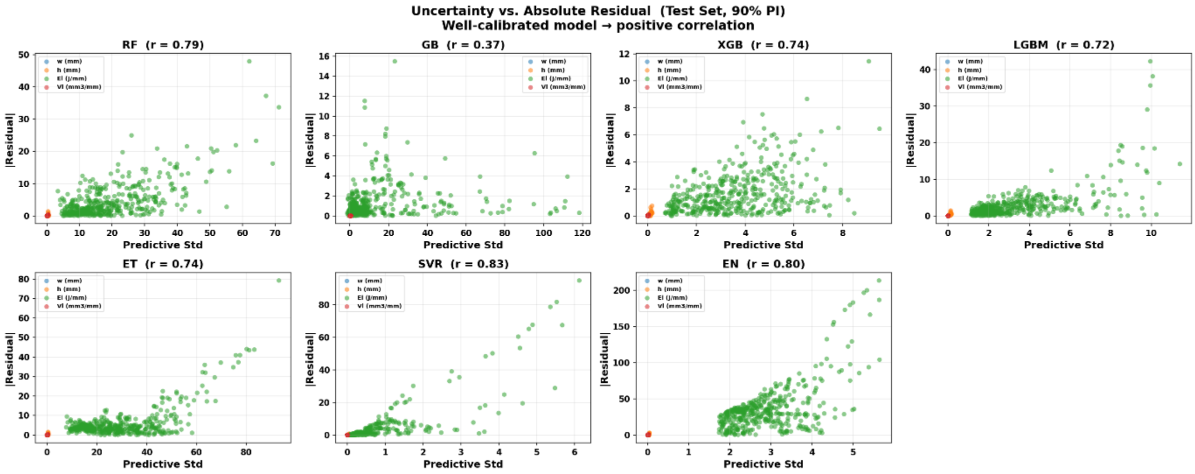

Figure S6. Uncertainty vs. Absolute Residual for 90% PI using test dataset.

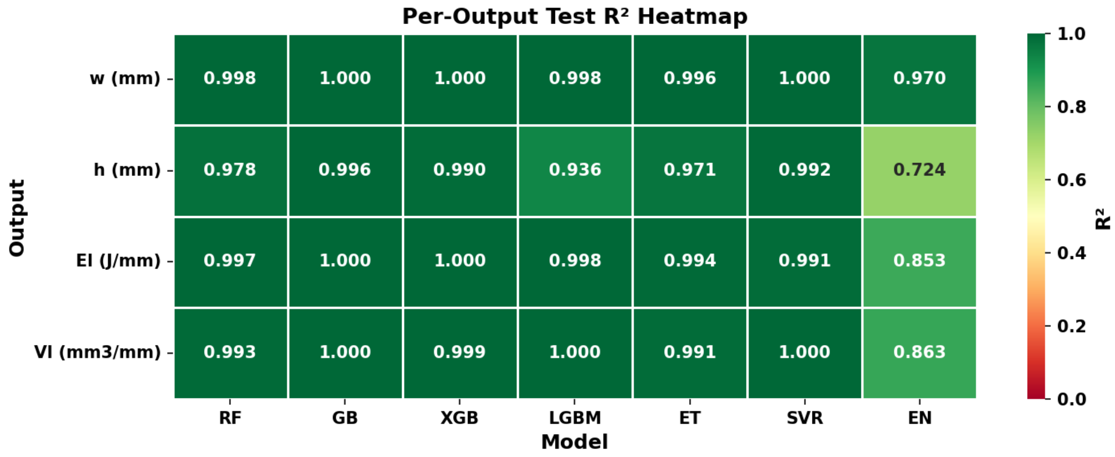

Figure S7. R2 values for each output using the test dataset for all used ML models.

Disclaimer/Publisher's Note: The statements, opinions and data contained in all publications are solely those of the individual author(s) and contributor(s) and not of MDPI and/or the editor(s). MDPI and/or the editor(s) disclaim responsibility for any injury to people or property resulting from any ideas, methods, instructions or products referred to in the content.
